# Parotitis-Like Symptoms Associated with COVID-19, France, March–April 2020

**DOI:** 10.3201/eid2609.202059

**Published:** 2020-09

**Authors:** Jerome R. Lechien, Annaelle Chetrit, Younes Chekkoury-Idrissi, Lea Distinguin, Marta Circiu, Sven Saussez, Najete Berradja, Myriam Edjlali, Stephane Hans, Robert Carlier

**Affiliations:** Foch Hospital, Paris, France (J.R. Lechien, Y. Chekkoury-Idrissi, L. Distinguin, M. Circiu, S. Hans);; University of Mons, Mons, Belgium (J.R. Lechien, S. Saussez);; University Hospital of Brussels (Saint-Pierre), Brussels, Belgium (J.R. Lechien, S. Saussez);; Hôpitaux R. Poincaré–Ambroise Paré, Paris (A. Chetrit, N. Berradja, M. Edjlali, R. Carlier);; Centre Hospitalier Sainte-Anne, Paris (M. Edjlali)

**Keywords:** parotid, parotitis, node, neck, France, COVID-19, coronavirus disease, SARS-CoV-2, severe acute respiratory syndrome coronavirus 2, viruses, respiratory infections, zoonoses

## Abstract

We report the clinical features of 3 patients in France who had parotitis (inflammation of the parotid salivary glands) as a clinical manifestation of confirmed coronavirus disease. Results from magnetic resonance imaging support the occurrence of intraparotid lymphadenitis, leading to a parotitis-like clinical picture.

The worldwide spread of coronavirus disease (COVID-19) is associated with the emergence of many clinical pictures of the disease. Patients might have nose and throat symptoms, such as loss of smell and taste (*1*). Many otolaryngologists have observed an increase in the number of patients with acute parotitis (inflammation of the parotid salivary glands), which could be related to COVID-19 (*2*). We report the clinical features of 3 outpatients who sought care at Foch Hospital (Paris, France) for parotitis-like symptoms in the context of COVID-19.

Three women sought care at the Department of Otolaryngology–Head and Neck Surgery of Foch Hospital for unilateral ear pain and retromandibular edema. The patients also reported general and otolaryngologic symptoms, including anorexia, arthralgia, myalgia, headache, fatigue, nasal obstruction, rhinorrhea, postnasal drip, sore throat, face pain, and loss of smell and taste (Table). Diagnosis of COVID-19 was confirmed by reverse-transcription PCR tests on nasopharyngeal swab specimens. The patients had no notable medical histories, and they were all vaccinated against mumps. The parotitis-like symptoms occurred at the onset of the disease in 2 patients and over the clinical course of the disease in the remaining patient. A clinical diagnosis of parotitis was made in all 3 cases. The otolaryngologist did not see any pus draining from the parotid duct. 

**Table:** Demographic, clinical, and imaging characteristics of 3 patients who sought care for parotitis-like symptoms associated with coronavirus disease, Paris, France, March–April 2020*

Patient age, y/sex	Symptom type			Duration of symptoms	Treatment	MRI diagnosis
	General	ENT	Parotitis			
23/F	Anorexia, arthralgia, myalgia, fatigue, headache	Nasal obstruction, rhinorrhea, postnasal drip, sore throat, face pain, loss of smell and taste	Ear pain, retromandibular edema	10 d	Paracetamol	Intraparotid, lymphadenitis
31/F	Cough, arthralgia, myalgia, fatigue, headache, diarrhea, abdominal pain, urticaria, dyspnea	Nasal obstruction, rhinorrhea, postnasal drip, sore throat, face pain, loss of smell and taste	Ear pain, retromandibular edema, sticky saliva, pain during chewing	15 d	Paracetamol, vitamins	Intraparotid, lymphadenitis
27/F	Cough, fever, anorexia, arthralgia, myalgia, headache, fatigue	Rhinorrhea, face pain, sore throat, loss of smell	Ear pain, retromandibular edema	3 d	Paracetamol	Intraparotid, lymphadenitis

*Diagnoses were made in Foch Hospital (Paris, France) on the following dates: March 21 (patient 1), March 27 (patient 2), and April 2 (patient 3). ENT, ear, nose, and throat; MRI, magnetic resonance imaging.

Patients underwent magnetic resonance imaging (MRI), which indicated intraparotid lymphadenitis. In all three cases, we observed multiple unilateral or bilateral intraglandular lymph nodes in the deep and surface layers, in a relatively normal-sized gland. We preserved the lymph node architecture by using a preserved fatty hilum. We observed no juxtaglandular fat infiltration or thickening of the fascia. We also observed no intraglandular linear bands or cysts on the MRI (Appendix). 

The 3 patients received 10–14 days of paracetamol (1 g 3–4×/d) for their COVID-19. The parotitis resolved over the next few days after diagnosis. The 3 patients had persistent loss of smell after the resolution of their general and parotitis-like symptoms.

The occurrence of acute parotitis related to COVID-19 has been suggested in a recent case report (*2*), corroborating the clinical observations of otolaryngologists. Our findings support the hypothesis that the parotitis-like symptoms might be attributable to intraparotid lymph node enlargement, which is different from a primary parotitis.

Infection with rubella, herpes, influenza, and human immunodeficiency viruses can result in salivary tropism (*3*,*4*), leading to diffuse parotitis. Our MRI findings mainly report diffuse enlargement of the gland without evidence of multiple intraglandular lymph nodes; however, the literature remains limited because the diagnosis is clinical and MRI is not often required. Mumps-related parotitis usually occurs in children and might be bilateral (*4*). In a patient with HIV infection, the parotid lesions appear as multiple and bilateral parotid lymphoepithelial cysts, which are bigger than lymph nodes (*5*). Moreover, cysts have T1 (hypo) and T2 (hyper) signals that are similar than those of the cerebrospinal fluid (*5*). In our patients, the MRI results did not indicate cysts. 

The features we describe support the diagnosis of adenitis, which might impair the gland functioning. The adenitis and the parotid-related enlargement might block the main gland duct (Stenon’s duct), leading to saliva retention and parotid tissue inflammation. The lack of saliva might be associated with sticky saliva and taste impairment. Intraparotid adenitis differs from primary diffuse parotitis, which was recently reported in an unique case of COVID-19 (*2*).

Sanitation conditions and the difficulties in performing additional salivary gland examinations (e.g., sialography or MRI) complicate the characterization of parotitis. Thus, the main limitation of our report is the lack of functional examinations of the parotid during the clinical course of the disease. The assessment of the functioning of the saliva secretion and the detection of severe acute respiratory syndrome coronavirus 2 virus (SARS-CoV-2) in the saliva could provide further information about SARS-CoV-2 transmission through saliva.

Future studies are needed to characterize the parotid manifestations in COVID-19 patients. Although the findings of this study support the hypothesis that intraparotid lymphadenitis is a causal factor, the direct spread of SARS-CoV-2 into the parotid tissue might be theoretically possible regarding the presence of angiotensin converting enzyme 2 (the virus receptor) in the parotid tissue and the potential risk for excretion of virions through the saliva (*6*).

In conclusion, parotid inflammation might be encountered in COVID-19 patients and could be related to intraparotid lymphadenitis. Even in persons vaccinated against mumps, testing for viruses that cause a parotitis-like illness is important, including rubella virus, influenza virus, and SARS-CoV-2. Additional studies to characterize parotid manifestations in COVID-19 patients will help determine diagnosis and treatment.

Appendix Additional information about parotitis-like symptoms associated with COVID-19, France, March–April 2020.
