# Supplementary material for: Parotitis-Like Symptoms Associated with COVID-19, France, March–April 2020
Source: Emerg Infect Dis. 2020 Sep;26(9):2270–1. doi: 10.3201/eid2609.202059 (PMC7454100; doi:10.3201/eid2609.202059)
Supplement: Appendix — Additional information about parotitis-like symptoms associated with COVID-19, France, March–April 2020. [file 20-2059-Techapp-s1.pdf]

Article DOI: https://doi.org/10.3201/eid2609.202059

Parotitis-Like Symptoms Associated with COVID-19, France, March–April 2020

Appendix

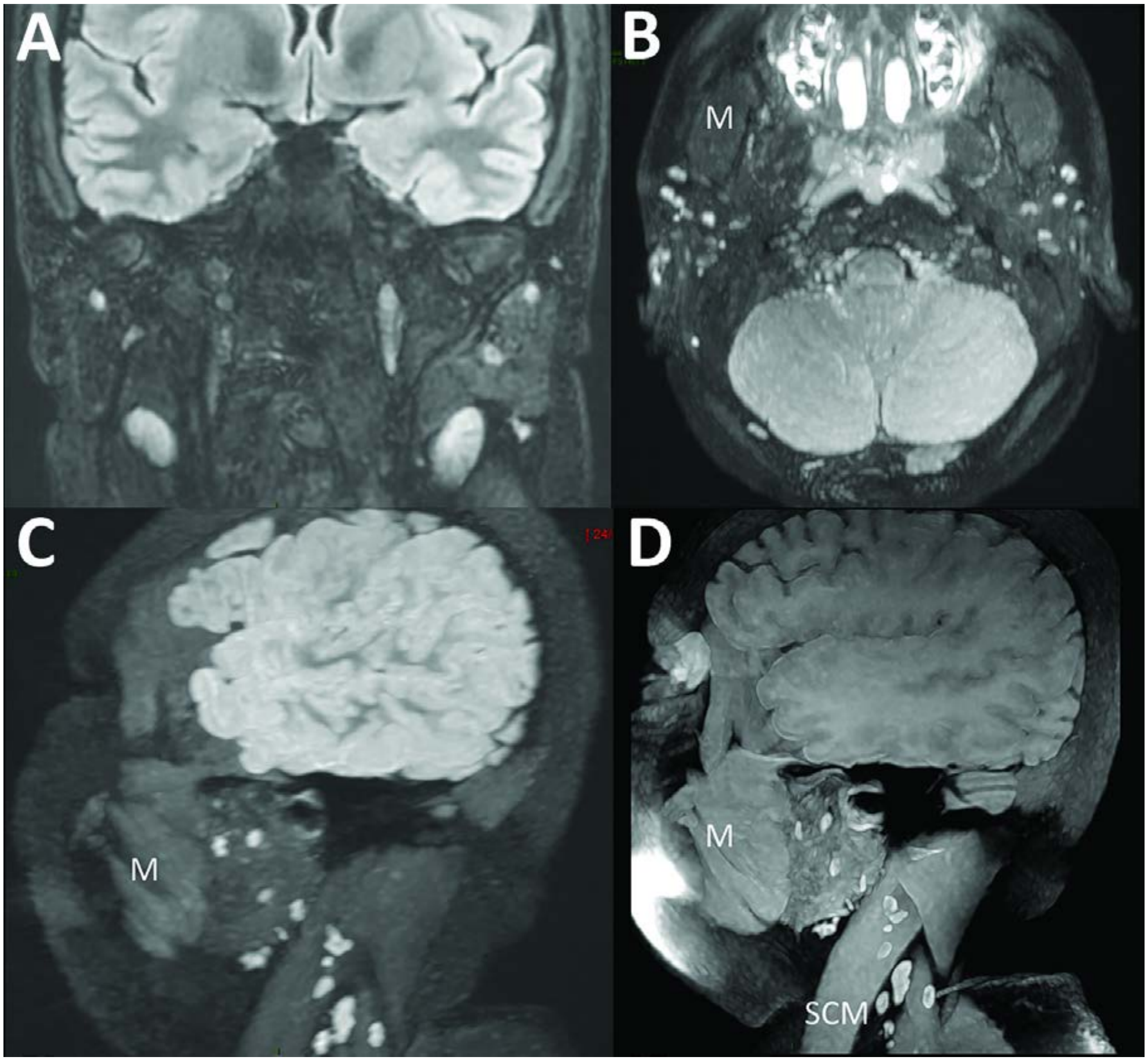

Appendix Figure. Magnetic resonance imaging of 3 patients who sought care for parotitis-like symptoms associated with coronavirus disease, Foch Hospital, Paris, France, March–April 2020.
